# Biology, Genetics, and Management of Ergot (*Claviceps* spp.) in Rye, Sorghum, and Pearl Millet

**DOI:** 10.3390/toxins7030659

**Published:** 2015-02-25

**Authors:** Thomas Miedaner, Hartwig H. Geiger

**Affiliations:** 1 State Plant Breeding Institute, University of Hohenheim, 70599 Stuttgart, Germany; 2 Institute of Plant Breeding, Seed Science, and Population Genetics,University of Hohenheim, 70599 Stuttgart, Germany; E-Mail: h.h.geiger@uni-hohenheim.de

**Keywords:** alkaloids, breeding, *Claviceps Africana*, *Claviceps fusiformis*, *Claviceps purpurea*, hybrid cultivar, *Pennisetum glaucum*, resistance, *Secale cereal*, *Sorghum bicolor*

## Abstract

Ergot is a disease of cereals and grasses caused by fungi in the genus *Claviceps*. Of particular concern are *Claviceps purpurea* in temperate regions, *C. africana* in sorghum (worldwide), and *C. fusiformis* in pearl millet (Africa, Asia). The fungi infect young, usually unfertilized ovaries, replacing the seeds by dark mycelial masses known as sclerotia. The percentage of sclerotia in marketable grain is strictly regulated in many countries. In winter rye, ergot has been known in Europe since the early Middle Ages. The alkaloids produced by the fungus severely affect the health of humans and warm-blooded animals. In sorghum and pearl millet, ergot became a problem when growers adopted hybrid technology, which increased host susceptibility. Plant traits reducing ergot infection include immediate pollination of receptive stigmas, closed flowering (cleistogamy), and physiological resistance. Genetic, nonpollen-mediated variation in ergot susceptibility could be demonstrated in all three affected cereals. Fungicides have limited efficacy and application is weather dependent. Sorting out the sclerotia from the harvest by photocells is expensive and time consuming. In conclusion, molecular-based hybrid rye breeding could improve pollen fertility by introgressing effective restorer genes thus bringing down the ergot infection level to that of conventional population cultivars. A further reduction might be feasible in the future by selecting more resistant germplasm.

## 1. Introduction

Ergot is the common name for a disease of grass inflorescences caused by fungi of the genus *Claviceps*. The term “ergot” also refers to the dark fungal structure produced within the floret and known as sclerotium. The genus *Claviceps* consists of a unique group of species, infecting only the ovaries of grasses and leading to sclerotia. Because *Claviceps* species are not able to penetrate through closed glumes, cross-pollinated crops are most threatened. There are approx. 40 species of *Claviceps*. The species of greatest concern is *Claviceps purpurea* (Fries ex Fries) Tulasne. It is distributed across most temperate regions of the world [1]. *Claviceps purpurea* has a very broad host range with approx. 400 grass species, including the cereals and all of the forage grasses in the temperate regions. Economically most important is the infection of rye (*Secale cereale* L.) which is a major crop in Germany, Scandinavia, Poland, Russia, Belarus and Ukraine [2].

Other *Claviceps* species mostly occur in tropical or subtropical areas and nearly all are restricted to a single host genus or closely related genera. Sorghum ergot caused by *Claviceps africana* Frederickson, Mantle and De Milliano is widespread in all sorghum (*Sorghum bicolor* Mönch) growing areas [3]. This species was formerly restricted to Africa and Asia where it was first recorded more than 90 years ago. In the mid-1990s, the Sorghum ergot spread to Brazil, South Africa, and Australia [3]. By 1997, the disease had spread to most South American countries and the Caribbean including Mexico, and reached Texas, USA, in March 1997 [4]. By October 1997, this ergot was already found throughout Texas and recorded in Georgia, Kansas, and Nebraska illustrating the high parasitic fitness of the species. Two other species causing sorghum ergot are *C. sorghi* Kulkarni, Seshadri and Hegde, first observed in India in 1915 and now widely distributed in eastern, western, and southern Africa, and *C. sorghicola* Tsukib, Shiman. and T. Uematsu known until now only from Japan, where it overlaps in occurrence with *C. africana* [3,5]. *Claviceps fusiformis* Loveless is widespread in Africa and India, where the host crop, pearl millet (*Pennisetum glaucum* (L.) R. Br.), has been grown for thousands of years. Sorghum is generally self-fertilized, pearl millet is predominantly outcrossing [6], and rye is strictly outcrossing [2].

The greatest threat from ergot is not the yield reduction of usually 5%–10% in commercial growing [7], but the contamination of the harvest by toxic alkaloids present in the sclerotia. *Claviceps purpurea* produces all three major groups of ergot alkaloids: Clavine alkaloids, D-lysergic acid and its derivatives, and ergopeptines; *C. africana* does not produce lysergic acid or derivatives thereof [8]. The alkaloids can cause severe health problems in both humans and animals. Up to the 19th century, prior to the introduction of grain standards for ergot, the sclerotia were ground up with rye grains and consumed because most of the flour was used for baking. Chronic consumption leads to symptoms that are summarized as “ergotism”. Strict thresholds are available in the European Union for soft and durum wheat with <0.05% by weight of sclerotia *i.e*., [500 mg·kg^−1^] for human consumption [9] that are also valid for rye in practical commerce. For animal feed, <0.1% of sclerotia is usually used as a threshold for all cereals. Similarly, in the USA, soft or durum wheat is regarded as “ergoty” when it contains more than 0.05% by weight of ergot. For barley, oat, and triticale more than 0.1% by weight of ergot sclerotia and for rye more than 0.3% by weight of scelerotia are thresholds [7]. Highly contaminated grain or spoilage from cleaning must be disposed of as hazardous waste.

Further problems with ergot arise with (1) commercial seed production for hybrid cultivars and (2) commercial growing of hybrid cultivars that are not fully restored, *i.e.*, poor pollen shedding. These are special problems connected with hybrid breeding on the basis of cytoplasmic-male sterility (CMS), that is nowadays widespread in rye, sorghum, and pearl millet. CMS plants are particularly vulnerable to ergot damage when flowering with the crossing partner is non-synchronous, resulting in a lack of pollen and delayed seed set. In hybrid sorghum production, losses up to 80% have been reported in India and Zimbabwe [3]. In grain harvested from hybrids, the percentage of ergoty grain might easily surpass the above-mentioned thresholds when the cultivars are not fully shedding pollen. In Germany, in the past, hybrid rye cultivars frequently had considerably more ergot sclerotia in the harvest than population cultivars when weather was favorable for the disease [10]. Similarly, in Africa or India, sorghum ergot caused by *C. africana* and ergot of pearl millet caused by *C. fusiformis* became economically significant only after the introduction of CMS-based hybrids [11,12].

CMS rye is successfully used to produce ergot sclerotia in the field for pharmaceutical purposes [13]. After artificial infection, the sclerotia yield reaches 1–2 tons per hectare. About 10–20 kg pure ergot alkaloids per hectare can be harvested [8]. Prerequisite is a specifically produced three-way hybrid composed only of maintainer lines in the CMS cytoplasm producing no pollen (=male sterile).

Several books are available covering either historical aspects of ergot e.g., [14,15,16] or biological, pharmaceutical, and toxicological aspects [17,18]. In this review, we are concentrating on management practices controlling ergot with a special emphasis on recent results in plant breeding.

## 2. History of Ergot in Europe

Ergot has always had a great impact on the rural people in Europe growing rye [19]. As a frequently occurring contaminant of the rye harvest, ergot sclerotia have caused poisoning over whole regions, causing a variety of symptoms referred to as ergotism. Two types of ergotism can be distinguished: “convulsive” and “gangreneous” ergotism [8]. The first type is characterised by muscle spasms, fever and hallucinations. The victims may appear dazed, be unable to speak, become manic, or have other forms of paralysis or tremors, and suffer from hallucinations and other distorted perceptions. This is caused by serotonergic stimulation of the central nervous system by some of the alkaloids. Human fertility can be reduced during ergotism outbreaks because women frequently miscarry. The second type is accompanied by violent burning, peripheral pulses and shooting pain of the poorly vascularized distal organs, such as the fingers and toes, and is caused by the potent vasoconstriction effects of some ergot alkaloids [8]. Continuing intoxication leads to the loss of peripheral sensation, edema and ultimately the dry gangrene and loss of affected tissues.

Outside Europe it was already mentioned in a sacred book of the Parsees [20] around 350 BCE, that “noxious grasses [that] cause pregnant women to drop the womb and die in childbed”. In the European Middle Ages, diseases caused by ergot were referred to as St. Anthony’s fire or “holy fire” [21]. Among the first documented epidemics of ergotism was that mentioned in the *Annales Xantenses* from 857 AD in western Germany and an epidemic in 944–945 AD from Limoges in France causing the death of approx. 10,000 people [16]. Some 50 years later, intoxication with ergot alkaloids was reported to have killed approx. 40,000 people in this area. Ergot alkaloid intoxication might have been connected with the witch trials of Salem, Massachusetts, USA, in 1692 [22] and in Finnmark, Norway, in the 17th century [23]. Although ergot was already used for medical purposes in the Middle Ages, it was obviously not recognized that ingestion of bread made from ergot-contaminated rye flour might pose a severe health risk. This was especially unfortunate for the poor farmers in Central and Eastern Europe, who depended on rye as their primary source for bread making [19]. Ergot was so commonly associated with rye that sclerotia were shown on the early botanical drawing of the plant from Caspar Bauhin in his book *Pinax Theatri Botanici* from 1658. A French physician, Thuillier, discovered the cause of ergotism in 1670. The disease could then be reduced by cleaning the grain from sclerotia before milling. Up to the early 19th century, however, thousands of people died of ergotism, and mortality rates reached up to 40% in some epidemics. Peter the Great could not proceed across the Volga River in 1722 on the way to Constantinople (Istanbul), because an ergot outbreak poisoned both his soldiers and their horses [19]. In 1853, Louis René Tulasne, a French mycologist, was the first to fully describe the life cycle of *C. purpurea*.

The earliest mention of medical use in Germany is in the herbal of Adam Lonicer from 1582, where ergot is mentioned as an aid in childbirth [16]. For centuries, midwives and medical doctors have used ergot extracts to accelerate childbirth or to induce abortions. Modern research on ergot alkaloids started in 1918 with the isolation of ergotamine by Arthur Stoll from Switzerland [24]. In 1926, Swiss psychiatrist Hans Maier suggested to use ergotamine for the treatment of vascular headaches of the migraine type [24], for which this alkaloid continues to be prescribed. Lysergic acid diethylamide (LSD) is a synthetic derivative of lysergic acid, a major component of ergot alkaloids. LSD was firstly synthesized by the Swiss chemist Albert Hofmann in 1938, while searching for a respiratory and circulatory stimulant. He discovered its effect on the nervous system accidently in 1943 when he ingested the substance and mentioned a hallucinogenic reaction [25]. The drug became popular in the mid-1960s because of its psychedelic properties. Currently, over a thousand compounds have been derived from ergot ingredients. There is hardly another source of natural products with greater value to the pharmaceutical industry. Drugs in neurology and psychology, for example, are still derived from ergot as well as those used in modern obstetrics.

## 3. Biology of Ergot Infection

**Figure 1:**
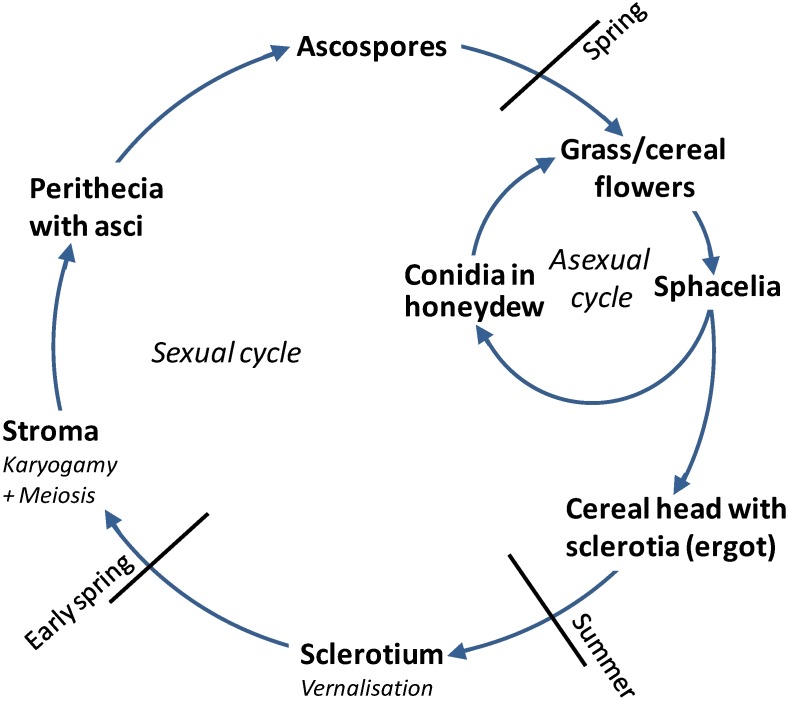
Life cycle of *Claviceps purpurea* (according to [19]).

The life cycle of *Claviceps purpurea* starts when windborne ascospores land on the featherlike stigmas of susceptible wild and forage grasses in the spring (Figure 1). The stigmas are efficient in trapping both pollen and ascospores [26], Figure 1). Ascospores are the primary (initial) inoculum germinating and infecting the ovary within 24 h. Hyphae invade and exclusively colonize the ovary, growing down to the tip of the ovary axis, the rachilla, and establishing a highly specific host-pathogen interaction mimicking pollination [27]. In the infected ovary, a spacelial stroma grows, producing masses of haploid, one-celled conidia which are exuded into a sticky, syrup-like fluid, called “honeydew”. Honeydew attracts insects, especially flies and moths. As these insects transfer honeydew to other flowers, they contribute largely to disease spread. Additionally, the honeydew can be transferred by rain splash, head-to-head contact or farming equipment. Honeydew production continues till the formation of sclerotia starts. Sclerotia mature within four to five weeks, replacing the seed.

Cereals are mainly infected by secondary inoculum*. i.e.*, conidia from ergot-infected wild grasses growing in or near the fields. Generally, resistance to *C. purpurea* develops a few days after fertilization. All conditions delaying pollination, such as cool, wet weather, increase, and therefore, so does the period of susceptibility. The infection process is repeated on the cereals like described for the forage and wild grasses. In rye, sclerotia can be up to 4–5 cm long, multiple sclerotia can be found on the same head. The sclerotium consists of a whitish mycelial tissue containing storage cells and a dark pigmented outer cortex that protects the fungal mycelium from desiccation, UV light and other adverse environmental conditions. Sclerotia are the survival/overwintering structures of *Claviceps* [27].

At harvest, a fraction of the sclerotia is harvested together with the grain, another fraction falls on the ground during threshing and remains on the soil at the end of the season. Temperatures at 0 °C–10 °C for at least 25 days [28,29] are required for vernalization of the sclerotia. Sclerotia lying above or just beneath the soil surface germinate in the spring, just prior to flowering of grasses, and give rise to one to several stromata, formed in mushroom-like fashion on stipes (stalks) with spherical capitula [27]. Female ascogonia and male antheridia develop in the periphery of the capitula and fuse (=karyogamy) to give for dikaryotic ascogeneous hyphae. After meiosis sexual fruiting bodies, the perithecia are produced. Perithecia are filled with asci, each containing eight long and thin ascospores. Rainfall or high soil moisture stimulates stroma formation and ascospore production. In moist conditions, ascospores are ejected into the air providing primary inoculum.

The life cycle of *C. africana* has some striking differences from that of *C. purpurea*. The host range is much smaller including, besides *S. bicolor*, only some other sorghum species and pearl millet [3]. The sexual stage has not been confirmed to date and the whole disease cycle is thought to be completed with asexually produced conidia. Infected sorghum flowers exude honeydew with a high concentration of primary conidia, but additionally microconidia are produced that are windborne over moderate to large distances [30]. The disease is known for its rapid, uncontrollable spread. In Australia, in April 1996, the disease was observed over an area of approx. 16,000 km^2^ within one week, and after one month it had expanded to 70,000 km^2^ [3]. In contrast to *C. purpurea*, insect transmission plays no significant role in spreading sorghum ergot [3]. The next season starts with mature sclerotia mixed with seeds or infected panicles fallen on the ground during the foregoing harvest and/or honeydew remnants sticking to the seed. Dried honeydew stays infectious for 9–12 months [30]. *Claviceps fusiformis* and *C. sorghi* also produce microconidia. The perfect stage of the fungi causing sorghum ergot has only been detected for *C. sorghi* in India [3].

## 4. Genetic Variation within *Claviceps* spp.

*Claviceps purpurea* is morphologically a highly variable species with respect to sclerotial length and shape, color of the stromata, and conidial size and shape [1,31]. This also applies to alkaloid spectra, where different isolates can produce different types of alkaloids [31]. The genetic system controlling the sexual cycle is monoecism and self-compatibility, *i.e.*, inoculation of one isolate is enough to complete the whole lifecycle of the fungus [31].

Initially, no host-specific subgroups within the species *C. purpurea* were found by Campbell [32]. Darlington *et al.* reported that male-sterile wheat and barley lines could be infected by isolates from many grasses [33]. Isolates of *C. purpurea*, however, highly differed in their aggressiveness towards cereals ranging from 12%–63% infected male-sterile barley or wheat florets among 48 isolates tested. Geiger and Bausback [13] observed among three isolates smaller differences in aggressiveness on male-fertile than on male-sterile rye. Obviously, missing pollen facilitated infection by the less aggressive isolates.

Recently, molecular studies have revealed three habitat-specific groups of *C. purpurea* [34]: G1 from fields and open meadows, including all isolates from cereals, G2 from shady or wet habitats and G3 from *Spartina anglica* from salt marshes [1]. Group G3 was described as a new taxon, *C. purpurea* var. *spartinae* [35]. Pažoutová [36] hypothesized that G2 and G3 might have developed from a single clone and adapted to the life in extreme environments. Based on molecular markers or alkaloid types, no host specialization within the three groups could be found among 100 isolates [34]. Isolates were, however, very variable in their genotype as well as their composition and types of alkaloids that they produced. Within G1, no two isolates had an identical molecular fingerprint [37]. This points to a panmictic population that is reproducing mainly sexually, *i.e.*, epidemics start every year anew from ascospores.

*Claviceps africana* differs from *C. purpurea* on the population-genetic level. Among a worldwide collection of 140 isolates of *C. africana*, 15 haplotypes were detected, 13 of them from Africa [38]. Thus, Africa can be considered a center of diversity. Based on molecular analyses, asexual reproduction seems to be predominant in Africa. Asexual primary conidia in the honeydew and asexual secondary, airborne conidia are the main inoculum sources. The population found in Australasia contained two closely related haplotypes (E, Ea). All American isolates belonged to another single haplotype (W) which was also detected two times in South Africa [11]. Isolates from Brazil, Bolivia, Mexico, Puerto Rico and Texas, USA, shared the same molecular haplotype illustrating the spread of only one haplotype from Africa to the Americas where it is further reproducing asexually [11].

## 5. Factors Affecting Ergot Infection

For ergot infection, host flowering biology is of fundamental importance. Ergot mimics the pollination of a cereal for fertilization. After penetration of the pistil, the fungus grows down the style either according to the pollen tube path outside the ovule or laterally entering the ovary [39]. The ovary wall is completely colonized after some days, but the hyphae do not spread further into the tissue.

At least three mechanisms affect ergot susceptibility [40,41]:

● Floral characteristics affecting stigma receptivity, e.g., time the florets stay open, stigma size, start of stigma drying after pollination,
● Ability of a plant to pollinate and fertilize before infection occurs, mainly dependent on availability of pollen,
● Resistance that hinders fungal infection or fungal spread in the gynoecium.

The situation is further complicated because all of these mechanisms are highly affected by weather shortly before and at flowering. Complex interactions between weather, fungal infection and spread, and pollen availability must be considered.

### 5.1. Floral Characteristics

In sorghum, flowering characteristics affecting ergot susceptibility have been described in detail. In this pathosystem, ergot conidia cannot infect the stigmas of fertilized flowers, hence, only a short infection period exists [3,42]. The relative growth rate of pollen tube and of hypha determines whether ovaries will be fertilized by pollen or colonized by ergot. In particular, the following factors have been shown to be important for low ergot susceptibility: (1) small size of stigmas; (2) short duration of stigma receptivity including particularly (i) less aperture at glume tip; (ii) short exposure time of stigma before pollination; (iii) rapid stigma drying after pollination [43]. In a detailed case study, a restorer line in CMS cytoplasm was described that was highly susceptible to ergot in spite of being a good pollen shedder, because the stigmas emerged from glumes two to three days before anthesis, thus prolonging the time span for infection.

In rye, the influence of flower characteristics has not been analyzed so far. However, it has been shown that *C. purpurea* can infect already pollinated ovaries [28]. On the other hand, receptive florets only open for a short period and close tightly after pollination. Stigmas then wither quickly. Opening of florets starts in the middle of the ear and progresses over two to three days. Larger rye fields composed of millions of single ears are in bloom for one to two weeks. Late tillers may flower even after this period providing an ideal infection target for ergot spores. Synchronous flowering among parent lines in hybrid seed production, and a short period of time when open flowers are available for infection in commercial stands surely reduce ergot infection.

### 5.2. Pollen Availability and Weather

Because ergot can most efficiently infect florets that are not yet pollinated or are pollinated shortly beforehand, competition occurs between ergot infection and pollination and a reduced amount of pollen favors ergot infection [10,44]. This is especially the case, (1) when a hybrid cultivar is not fully restored to fertility; (2) during hybrid seed production, when the flowering times of the seed and pollen parent are not fully synchronized; or (3) generally when weather conditions are cool and rainy during flowering.

Unfavorable temperatures and high humidity around the time of meiosis, *i.e.*, some days before pollination, negatively affects pollen production and viability. Ergot infection of sorghum by *C. africana* is favored by cold night temperatures (<12 °C) 2–3 weeks before flowering inducing pollen sterility of the host, or cool (*ca.* 20 °C), moist weather during flowering [11].

In all crops, rainy weather at flowering time reduces pollen shedding and pollen movement. The wet pollen agglutinates and is distributed over short distances only. Thus, many florets remain open longer because they are not readily fertilized. In rye, unfertilized florets can re-open for several days. During this time, the stigma grows longer and, thus, increases its surface for more efficiently trapping pollen and ergot spores. Cool and rainy conditions also favor the spread of honeydew by insects or wind promoting the proliferation of the pathogen and increasing infection frequency. Warm and sunny conditions, in contrast, thicken the honeydew and lead to a short time of stigma receptivity thus reducing ergot infections.

Temperature and humidity also affect fungal growth in the stigma as compared with that of the developing plant embryo. *Claviceps purpurea* can grow over a wide temperature range, but the optimum is between 20 °C and 30 °C [45]. Below 10 °C germination and subsequent mycelium development are highly retarded. Above 10 °C, vigor and rapidity of growth are directly correlated to temperature, at least in saprophytic *in vitro* culture [45]. Consequently, low temperatures at flowering may reduce infection frequency and retard fungal growth, and the plant embryo gains a lead in development.

If genotypes largely differ in flowering date, host genotype-by-environment interaction effects may become important. Mean daily maximum temperatures one to five days after flowering have accounted for 31%–61% of variation in ergot resistance of sorghum [46]. In artificial infection studies with *C. purpurea* and male-sterile rye, host genotype-by-environment interaction had a higher impact than genotypic effects [41]. In male-fertile rye, this component of variation was significant (*p* < 0.01) as well [47,48]. This underlines the necessity to test over several locations and years. Consequently, the ranking of genotypes in one test environment might not be closely correlated with other environments or a specific test environment might not discriminate the genotypes enough to find full genetic variance [49].

### 5.3. Host Resistance

Host resistance may be based on passive or active mechanisms or a combination of both. The most effective passive mechanism is avoidance by cleistogamy. Self-pollinating crops, like wheat, barley and sorghum, where the florets generally do not open, have only a low ergot incidence. For outcrossing crops, like rye and pearl millet, and for the hybrid production of both self- and cross-pollinating crops, flowers must open wide to ensure cross pollination. In pearl millet, lines with a shorter period of protogyny displayed lower ergot susceptibility, because the stigmas become unreceptive before the fungus can infect [50]. This passive mechanism (“disease escape”) was further enhanced by localized stigmatic constriction [6]. However, when the constriction occurs early outcrossing is hampered, a feature that puts hybrid seed production at risk.

Active mechanisms of resistance caused by gene-for-gene relationships (qualitative resistance) or by several genes with a minor effect of each gene (quantitative resistance) have not been found so far. When resistance was claimed, it remained open whether it simply was a consequence of flower morphology, flower habit or pollen availability. In rye, formation of callose in the ovaries has been suggested as a component of ergot resistance [51]. Moreover, it has even been speculated that ergot uses components of the signal exchange between pollen and stigma for its own benefit [27]. When the ergot fungus closely interferes with pollen growth, a resistance reaction would counteract fertility and thus no such resistance mechanism is likely to have arisen by evolution. The same applies to a hypersensitive necrotic response of the ovaries against ergot infections [52].

## 6. Breeding Strategies for Reducing Ergot Susceptibility

For improving disease resistance in plant breeding, three steps are required: (1) evaluation of genetic resources and selection of resistant germplasm; (2) crossing selected genotypes with elite breeding materials and evaluation of segregating progeny; and (3) selection of the most promising progenies and using the best ones as resistance donors in cultivar development.

### 6.1. Field Design for Testing Ergot Susceptibility

In plant breeding experiments, cultivars are generally grown side by side in a randomized field design with small plot sizes (1–10 m^2^). In tests for ergot resistance, pollen shedding of the neighboring plots will have a direct effect on the entry plots and may cause a strong inter-plot interference. When for example a poor pollen-shedding entry is grown between two plots with high pollen shedding, ergot severity will be much lower than when the same entry is grown between neighbors with less pollen shedding.

In Germany, three types of cultivars in rye are considered by the Federal Plant Variety Office: Open-pollinated populations, synthetics and hybrids [53]. Hybrid cultivars are exclusively in CMS cytoplasm and their pollen fertility ranges from low to almost normal, synthetic cultivars may contain male-sterile, CMS-based plants in varying percentages, and population cultivars are exclusively composed of plants in normal cytoplasm displaying full pollen shedding.

When testing for ergot resistance, the field design needs to account for the male-fertility level of the entries. In this regard, there are three types of entry in hybrid breeding nurseries:

1. Fully pollen shedding (e.g., landraces, population cultivars, fully male fertile inbred lines),
2. Varying degrees of pollen shedding (e.g., hybrids, synthetics, partially male fertile inbred lines),
3. No pollen shedding due to complete male sterility (e.g., CMS lines, seed parent single crosses).

Plant materials included in class (1) need no special design, *i.e.*, can be randomized without any restriction. Materials of class (2), however, need special test systems ensuring minimum inter-plot interference [48]. Entries of class (3) can be fully randomized if they are grown in strict isolation from other fields of the same crop.

For materials of class (2), each plot should be neighbored by two border plots of the same size. Border plots could consist of another cereal crop or of the entry itself, *i.e.*, growing triple plots of each entry but harvesting the center plot only. Border or triple plots should additionally reduce physical contact between honeydew-bearing heads from neighboring plots.

Artificial inoculation is recommendable due to the unpredictable spread of natural inoculum strongly influenced by humidity. The use of large plots (5–8 m^2^) and a commercial spraying machine for inoculation further increase testing accuracy. Plots should be inoculated several times during flowering because heterogeneous genotypes usually flower at different dates and the period of maximum susceptibility is very short. Even then, ergot infections in rye may fail in those environments, where not enough humidity is available or where it is too cool during flowering. A high artificial disease pressure minimizes the risk of disease escape. However, not every location is optimal for selection. In a study by Dhillon *et al.* [49], a location with the highest mean ergot severity across years showed the lowest discrimination ability among entries and lowest prediction ability for other locations.

Ergot incidence (% of affected heads) and ergot severity (% of sclerotia in grain by weight) are the most representative resistance traits when using pollen-shedding material. In rye, both traits are highly correlated (*r* = 0.69–0.97) and have similar heritabilities (*h*^2^ = 0.90 and 0.89, respectively, [48]). The percentage of sclerotia in the grain, however, reflects the threshold value being controlled visually during marketing. Harvest of inoculation trials should be before maturity to reduce the risk of losing sclerotia caused by wind, rain, or thunderstorms. At harvest, a subplot of approx. 1 m^2^ from the middle of the center plot should be cut at approx. 30 cm below the main ears by hand. This avoids the inclusion of secondary or tertiary tillers in the harvest potentially bearing much more sclerotia than the main stand due to reduced pollen shedding at their late flowering times. Healthy and infected heads are visually separated, counted, and the percentage of affected heads is calculated for measuring ergot incidence. For assessing ergot severity, all heads per plot are dried, and threshed as one sample. Afterwards, all sclerotia or sclerotial fragments are picked out by hand from a random sample of 500 g, weighed, and the weight is calculated relative to the total sample weight.

### 6.2. Genetic Variation among Open-Pollinated Populations

In rye, large collections of fully pollen shedding landraces and old cultivars from Central and Eastern Europe and other countries exist in gene banks. Mirdita *et al.* [54] evaluated 245 rye entries derived from five base populations used for breeding purposes in Germany, Ukraine, and Russia (Table 1). Ergot severity ranged from 1.19%–8.07% sclerotia in grain by weight. Since no highly resistant germplasm was found in this population sample, a wider range of genetic resources and cultivars was tested. Again, no fully resistant material was detected, but significant quantitative differences existed among entries [47]. The range of ergot severities, however, was not larger than among released open-pollinated cultivars. Synthetic and hybrid cultivars used as standards showed a much higher ergot severity in the same experiment (Table 1) due to the occurrence of poorly restored plants shedding less pollen.

**Table 1:** Means and genotypic ranges of ergot severity measured as sclerotia in grain by weight (%) of different winter rye materials after inoculation by *Claviceps purpurea* across four environments (*N* = number of entries, OP = open pollinated).

Plant material	*N*	Ergot severity (%)	
		Mean	Range
Base populations ^a^	245	2.94	1.19–8.07
Genetic resources ^b^	52	2.31	1.36–4.32
Released OP cultivars ^b^	13	2.22	1.32–3.75
Synthetic cultivars ^b^	2	3.84	3.59; 4.08
Hybrid cultivars ^b^	2	7.54	6.46; 8.62

^a^ [54]; ^b^ [47].

Analyses of variance revealed high heritabilities despite high genotype–environment interactions. Thus, recurrent selection based on multi-environment infection trials should significantly improve the resistance level. Obviously, superior genotypes detected within released populations can directly be used as resistance donors in ongoing cultivar development.

### 6.3. Genetic Variation among CMS-Based Hybrids

Ergot may cause serious problems in growing hybrid cultivars and in hybrid seed production. In hybrid breeding, two or more preselected inbred lines of genetically divergent gene pools are used as parents of an F_1_ cross (hybrid). Hybrids greatly outperform their parental lines in cross-pollinating crops. Their superiority is called hybrid vigor or heterosis. Because heterosis is maximal only in the F_1_ generation, hybrid seed has to be produced every year from anew by crossing their parental inbred lines. In cereal crops where stamens and pistils are in the same floret, like in rye, sorghum and millets, CMS is the basis for hybrid breeding. CMS versions of the female parent that shed no pollen are used for producing the large amounts of seed needed by the farmer. To restore pollen production in the commercial hybrid, the male parent (pollinator) must possess special nuclear pollen-fertility restorer gene(s). Ideally, the hybrid is fully restored, shedding the same amount of pollen as a genotype in normal cytoplasm. However, in various present-day hybrids pollen-fertility restoration is considerably lower than 100%. The degree of pollen-fertility restoration depends on (1) the CMS cytoplasm used; (2) the genotype of the seed parent; (3) the effectiveness of the restorer gene(s); and (4) environmental conditions before and during flowering.

Ergot contamination of hybrid seed can be effectively reduced by opto-electronical color sorting machines. They separate the dark-purple ergot sclerotia from the lighter colored cereal grains. However, when the level of contamination is too high, seed production becomes economically unviable.

#### 6.3.1. Variation in Ergot Susceptibility of CMS Lines under Pollen Isolation

To test whether genetic differences for ergot susceptibility exist in completely male-sterile seed parent materials in rye, which would not be caused by differences in pollen availability, Miedaner *et al.* [41] tested a representative sample of 64 CMS lines in pollen-isolated plots under artificial infection with *C. purpurea*. Under these highly conducive conditions for ergot, practically no kernels are produced. Genotypic variance was highly significant for the weight of sclerotia per ear. Entry means ranged from 0.72–2.87 g. In corresponding F_1_ materials in CMS-inducing cytoplasm, genotypic variance was smaller and depended on the female as well as the male parent. This corroborates earlier results of Geiger and Bausback [13] who used a similar approach but fewer genotypes.

Significant differences in ovary colonization rates were found among three male-sterile sorghum lines after inoculation with *C. africana* in a controlled-environment facility [55]. In a follow-up study, Reed *et al.* [46] crossed each of five CMS-lines by five pollinator lines without pollen-fertility restorer genes and got a good genotypic differentiation in Puerto Rico and Mexico. On average, the pollinator lines had low (7%–10%) and the CMS female lines had very high (62%–82%) ergot severities as defined by percentage of infected florets per panicle. Hybrid means ranged from 27%–70%. Differences between hybrids could mainly be attributed to the general combining ability (GCA) effects of the male parents.

Dahlberg *et al.* [43] reported that Ethiopian gene bank accession IS 8525 has a high level of resistance to *C. africana* in the CMS cytoplasm. This line was crossed to a susceptible elite line and 289 F_5_ lines derived from this cross were tested [56]. Ergot severity varied quantitatively with a high heritability (*h*^2^ = 0.8). Pollen quantity and pollen viability accounted for only a small part of the genetic variation (11% and 9%, respectively). A companion analysis of quantitative trait loci (QTL) confirmed this finding. Parh *et al.* [40] detected nine, five and four QTL for ergot severity, pollen quantity and pollen viability, respectively. Co-localization of QTL for these traits was detected on four chromosomes while other QTL mapped independently. In this study, the mentioned pollen traits played only a minor role for ergot susceptibility. The potential effect of floral characteristics was not analyzed. It is known, however, that the resistance donor has a short, narrow stigma, no stigma exposure before anthesis, and the stigma rapidly dries after pollination [43]. Because these traits are highly heritable, accession IS 8525 is widely used as a resistance donor in breeding programs. The low susceptibility might, however, be caused more by the above-mentioned floral traits than by active resistance mechanisms.

Commercial hybrid breeding has been started in wheat, triticale, and barley in Europe [57], based on gametocides (wheat) or CMS (triticale, barley). CMS-based hybrids will suffer from ergot when restoration is not high enough [58]. It was shown earlier that unpollinated, hand-castrated wheat had a high susceptibility to *C. purpurea* with disease severities ranging from 16%–82%. Low susceptibility was mainly due to a rapid resistance development after pollination [59]. Likewise, in CMS wheat and barley, lines displayed large differences in ergot severity [33,58].

In summary, various experiments with rye, sorghum, wheat and barley CMS lines demonstrated that non-pollen mediated resistance to ergot is available, slowing down the spread of the fungus in the gynoecium. This should be used in selection to accomplish a higher level of resistance than solely by improvement of pollen-fertility restoration.

#### 6.3.2. Selection for Pollen-Fertility Restoration in Rye

**Figure 2:**
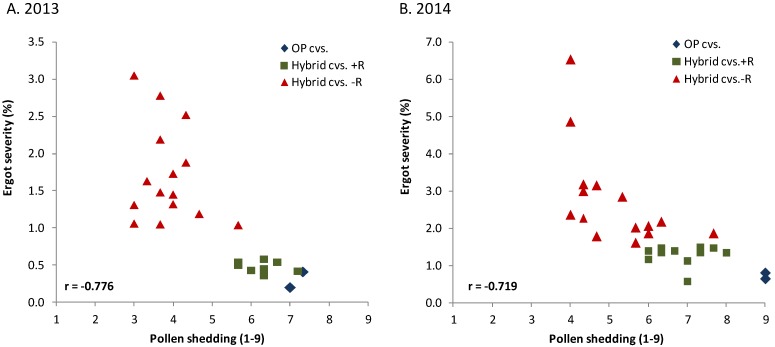
Relationship between ergot severity (% sclerotia in grain by weight) and pollen shedding (anther score 1–9 with 1–3 = no pollen shedding, 4–6 = partially restored, 7–9 = maximal pollen shedding) for open pollinated (OP) population cultivars (cvs.) and hybrid cultivars with (+) and without (−) a restorer gene (R) from a non-adapted source in field experiments with artificial infection by *C. purpurea* across five locations in each of two years (r = combined coefficient of correlation, *p* < 0.01).

Soon after the first release of hybrid rye cultivars in 1985, it was recognized that this new type of cultivar shed, on average, less pollen than population cultivars [10,60,61]. This could also be validated in more recent experiments, in which hybrid cultivars had an average ergot severity of 7.54% compared to 2.22% observed in released open-pollinated population cultivars (see Table 1). The anthers of hybrids were smaller [10] and contained less pollen [62]. In field experiments with artificial infection, hybrid cultivars partially restored (4–6 on a 1–9 scale) had, on average, a considerably higher ergot severity than population cultivars (Figure 2, hybrid cvs. −R).

The large difference within this group is mainly caused by the different ability of the female parents to be restored. Though blending hybrid seed with 10% seed from a population cultivar reduced ergot severity among the highly susceptible hybrids. It was, however, not effective enough to sufficiently control ergot severity in environments with high disease pressure [48].

The main cause for poor restoration of many rye hybrids is the low restoration ability of European restorer sources for the widely used Pampa cytoplasm from Argentina [63]. In the meantime, genes with higher restoration ability were detected in non-adapted genetic resources from Iran and Argentina and introgressed into elite pollinator germplasm. The introgressed genes raise pollen fertility to a much higher level (55%–90%) than former restorer genes found in adapted European cultivars (2%–74%, [64]). In 2005 and 2006, the first two hybrids (“Pollen Plus”) were listed in Germany both carrying a new, highly effective restorer gene derived from rye accession IRAN IX (P. Wilde, personal communication). Some hybrids with this gene meanwhile reach the same low level of ergot severity as population cultivars (Figure 2, Hybrid cvs +R). This clearly illustrates important progress in reducing the susceptibility of modern hybrids to ergot.

### 6.4. Genetic and Environmental Variation of Alkaloid Contents

Ergonovine (=ergometrine), ergotamine, ergocornine, ergocryptine, ergosine, and ergocristine are reported to be the main alkaloids of *C. purpurea* [65]. For each alkaloid, -inine isomers are regarded, however, as less toxic [66]. Isolates of *C. purpurea* differ highly in their composition of alkaloids, as controlled genetically and environmentally [67,68,69,70]. Total alkaloid concentration is used as a measure of toxicity (*cf.* [66]), because the toxicity of the individual alkaloids is not established.

Miedaner *et al.* [41] analyzed the content of the six mentioned alkaloids including their -inine isomers in sclerotia collected from 25 CMS rye inbred lines. Little or no genotypic variation for the concentration and composition of alkaloids in the sclerotia was found, although ergot severity among CMS lines significantly differed. Also, Mainka [66] previously found no association between total alkaloid concentration in the sclerotia and ergot severity of the rye genotypes. For breeding purposes, therefore, costly and time consuming alkaloid analyses are not necessary. The main target of the breeders should be reduction of ergot severity. However, we found a strong influence of the environment (location × year combination) on the concentration and even the relative proportion of individual alkaloids, although the same inoculum was used in all environments. The use of an inoculum composed of several isolates might have contributed to this result. In one year, the largest proportions had ergotamine (25% of total alkaloid content) and ergosine (18%), in the following year on the same location the largest proportion was found for ergosine (25%) and ergocristine (24%), whereas the ergotamine concentration reached only 11%. Yet, considering the large effect of the environment, grain samples from different locations or years may considerably differ in the concentration and composition of their alkaloids while still meeting the legal threshold of percentage sclerotia.

## 7. Management Factors

Besides growing host cultivars with a low ergot susceptibility, various other management factors will reduce ergot contamination of the grain from commercial fields.

### 7.1. Agronomic Measures

Dense cereal stands: Late tillers and side shoots flowering outside the pollination period of the main stand are more affected by ergot than main shoots, especially, when the cereal stand is thinned out by unfavorable agronomic conditions [7]. Late tillers show much more sclerotia than the primary ears because they usually do not receive enough pollen. Late infected spikes may still produce honeydew although the main crop is already mature, thus contributing considerably to the ergot load of the harvested grain. Therefore, the best crop husbandry against ergot is to care for a well-developed, well-nourished cereal stand, in particular by applying an adequate seed density and nitrogen fertilization. A closed canopy reduces the flowering span of the cereal stand and the production of late tillers. If ergot infection concentrates on the borders of the field, these should be harvested separately to reduce the ergot load of the whole field [7].

Crop rotation: Because ergot sclerotia usually do not survive longer than a year in the soil [19], rotation with a non-susceptible crop, for example a self-pollinating cereal, may strongly reduce infection pressure.

Deep plowing buries sclerotia in the soil. Ascospores are not formed or they cannot be released into the free air in spring [19]. When cereals are grown with “no-till” soil management, *i.e.*, new crops are grown directly in the residues of the previous crop, crop rotation is even more important.

Control of wild and weedy grasses and cereal volunteers: Wild and weedy grasses within or outside the fields are the first source of ergot inoculum in early spring from overwintered sclerotia, or as a source of honeydew if produced prior to crop flowering [26]. They should be controlled by herbicides along fences and hedges or by simply cutting grasses at the boundary ridges before flowering. Insects carry honeydew to cereal plants in the field and start new infection cycles. Similarly, cereal volunteers, especially rye plants in a stand of wheat or barley, will promote ergot because of the lack of pollen.

Irrigation: Information on the effect of irrigation practices is scarce. Because ergot develops most successfully under humid conditions, an irrigation at the beginning of flowering might promote the onset of the disease [71].

Burning: Post-harvest field burning is a proven measure to reduce inoculum from ergot and other diseases and pests [19]. Routine burning of fields is illegal in the USA and EU.

Certified seed: Certified seed contains ergot at the lowest level due to governmental control of seed multiplication. In the UK and Germany, only one sclerotium or one part of a sclerotium are allowed in 500 g basic seed of all cereals. Maximal three sclerotia or sclerotial pieces are tolerated in 500 g certified seed. Only hybrid rye is allowed to contain up to four sclerotia in 500 g certified seed in Germany. In India, the limit for certified seed of sorghum has been regulated at 0.04% ergot sclerotia relative to the number of seeds [3].

### 7.2. Fungicides

In Germany, no fungicides are officially recommended for control of *C. purpurea* in cereals although some strobilurins prevent germination of conidia, and tebuconazole and other azoles reduce mycelium growth of ergot by 70%–90% (Rodemann, pers. commun.). In Zimbabwe, benomyl at 0.2% a.i. reduced ergot infection in sorghum and in Brazil, propiconazole and tebuconazole applied in three to four sprays at 250 g·a.i.·ha^−1^ has successfully been used in seed production of sorghum [3]. Generally, control by fungicides is most effective with low inoculum pressure, under dry conditions and when spraying occurs before the onset of disease (preventive). However, the necessity of several sprays starting before cereal anthesis makes fungicide application in farmers’ fields economically unviable [71]. Moreover, the fungicide does not move systemically from head tissues into the stigma; therefore, thorough spray coverage of the head or panicle, directly wetting the stigmas, is essential [3].

In the USA, sterol-inhibiting fungicides were sprayed in forage and turf grass seed production routinely [19]. They can reduce the severity of ergot in Kentucky bluegrass, when applied at the onset of flowering [3].

### 7.3. Mechanical Cleaning

Removing sclerotia from grain before milling is a standard procedure in large mills. Several devices are possible; e.g., a gravity table, aided by an air stream separates sclerotia of lower specific gravity than grain. In sorghum, seeds are often sticky from honeydew, which makes mechanical cleaning difficult, so it has been recommended to wash seeds with large water volumes and dry them afterwards [3].

Optical-electronic color sorters separate cereals including rye from items with different color. This is especially successful for sorting the purple-black ergot from blueish-gray rye or, even better, light colored wheat or barley. The kernels pass a high-speed camera and, with pneumatic ejection devices, the unwanted ergot sclerotia are sorted out. However, costs of such equipment are high and the process greatly reduces flow capacity during milling. For commercial hybrid seed production in Europe, these devices are obligatory to fall below the threshold for certified seed.

## 8. Conclusions and Future Outlook

Ergot infections of cereals are a severe problem of food security. The toxicity of the sclerotia in the harvest, especially for young children and pregnant women, requires high standards of cleaning and sorting. Likewise, in animal feeding, ergot may become a problem when the feeding lots are contaminated. Due to the long co-evolution between cereal hosts and *Claviceps* spp., the fungus has developed subtle ways of infection, probably by mimicking pollen-tube growth. It is, therefore, not easy for the host to develop active resistance mechanisms. Passive avoidance of infections by cleistogamy is not an option for cross-pollinating crops or CMS-based hybrid crops of self-pollinators. In pearl millet and sorghum, however, some genotypes with a shorter or missing period of protogyny and a stigma rapidly drying after pollination showed a considerably lower ergot susceptibility and are now widely used for breeding. In hybrid crops, full pollen-fertility restoration is an effective way for reducing ergot contamination. Significant genetic variation is available for ergot resistance as shown by CMS materials. However, if resistance is inherited quantitatively, they are prone to genotype-by-environment interaction and gain from selection most probably will be slow. For both selection of effective restorer genes and of genes mediating host resistance, the use of molecular markers will be advantageous. Genomic selection procedures based on modern high-density single nucleotide polymorphism (SNP) assays should be the method of choice to improve complex traits like ergot resistance.
